# Impact of Three Oral Antidiabetic Drugs on Markers of β-Cell Function in Patients with Type 2 Diabetes: A Meta-Analysis

**DOI:** 10.1371/journal.pone.0076713

**Published:** 2013-10-25

**Authors:** Jin Lu, Jiajie Zang, Huihua Li

**Affiliations:** 1 Department of Endocrinology, Changhai hospital, Second Military Medical University, Shanghai, China; 2 Department of Nutrition Hygiene, Shanghai Municipal Center for Disease Control and Prevention, Shanghai, China; Broad Institute of Harvard and MIT, United States of America

## Abstract

**Background:**

The effect of metformin, pioglitazone and sitagliptin on β-cell function in the treatment of type 2 diabetes is controversial. Therefore, we performed a systematic review and meta-analysis to obtain a better understanding in the β-cell effects of metformin, pioglitazone and sitagliptin.

**Methods:**

We searched Pubmed and the Cochrane Center Register of Controlled Trials to identify relevant studies. Trials investigating effects of sitagliptin, metformin or pioglitazone on β-cell function were identified. The primary outcomes were homeostasis model assessment of β-cells (HOMA-β) and proinsulin/insulin ratio (PI/IR). Secondary outcome was hemoglobin A1c level. We used version 2 of the Comprehensive Meta Analysis software for all statistical analyses.

**Results:**

Metformin monotherapy was more effective than sitagliptin in improving HOMA-β (18.01% (95% CI 11.09% to 24.94%) vs. 11.29% (95% CI 9.21% to 13.37%), *P* = 0.040) and more effective (−0.137 (95% CI −0.082 to −0.192)) than both sitagliptin (−0.064 (95% CI −0.036 to −0.092), *P* = 0.019) and pioglitazone (−0.068 (95% CI −0.044 to −0.093), *P* = 0.015) in decreasing PI/IR. Metformin and sitagliptin combined (40.23% (95%CI 32.30% to 48.16%)) were more effective than sitagliptin and pioglitazone (11.82% (95% CI 6.61% to 17.04%), *P* = 0.000) and pioglitazone and metformin(9.81% (95% CI 1.67% to 17.95%), *P* = 0.022) in improving HOMA-β and decreasing PI/IR (−0.177 (95% CI −0.118 to −0.237); −0.080 (95% CI −0.045 to −0.114), *P* = 0.007; −0.038 (95% CI, −0.005 to 0.071), *P* = 0.023).

**Limitations:**

The included RCTs were of short duration (12–54 weeks). We could not determine long term effects on β-cells.

**Conclusions:**

Metformin improves β-cell function more effectively than pioglitazone or sitagliptin in type 2 diabetes patients. Metformin and sitagliptin improved HOMA-β and PI/IR more than other combinations.

## Introduction

Progressive β-cell dysfunction and failure are fundamental pathogenic consequences of type 2 diabetes. Diminished β-cell function on diagnosis and sustained decline in β-cell mass and function in type 2 diabetes suggest that medical therapies targeting pathogenic β-cell deterioration are needed. Assessment of β-cell function may be helpful in selecting the most appropriate therapy.

Both β-cell function, via the homeostasis model assessment of β-cells (HOMA-β) [1], and β-cell dysfunction, via the proinsulin/insulin ratio (PI/IR), can be measured in type 2 diabetes [2]. Increases in HOMA-β indicate preservation of β-cell function, whereas decreases in PI/IR driven by proinsulin indicate improvement in the secretory and, possibly, resistance profile of β-cells [3], [4]. Worsening of these surrogate measures may be correlated with β-cell failure. Reductions in HOMA-β have historically been a measurement of β-cell deterioration associated with both hemoglobin A1c (HbA1c) elevation and additional therapy to maintain glycemic goals, whereas increases in HOMA-β are considered beneficial [5]. PI/IR is negatively correlated with insulin secretion in both healthy subjects and those with diabetes, implying that PI/IR may be a strong predictor of β-cell dysfunction [6].

After the onset of type 2 diabetes, patients are usually treated via lifestyle modifications with or without different combinations of oral drugs. Studies have indicated that metformin, pioglitazone and sitagliptin may improve β-cell function [7]–[9]. However, the effectiveness of these glcose-lowering drugs on β-cell function in the treatment of type 2 diabetes remains controversial. Considering the lack of concordance among randomized trial results, we conducted a meta-analysis of randomized controlled trials (RCTs) published through September, 2012 to evaluate the effect of these glucose-lowering drugs on HOMA-β and PI/IR.

## Materials and Methods

### Data sources and searches

A literature search of PubMed (1976 onward) and the Cochrane Center Register of Controlled Trials (no date restriction) was performed using the medical subject headings *sitagliptin*, *metformin*, *pioglitazone*, *HOMA-β* and *proinsulin/insulin ratio*. A manual search was conducted of abstracts presented between 2003 and 2012 at the American Diabetes Association Scientific Sessions. We also reviewed the reference lists of the original and review articles to identify relevant studies. The search was initiated in September, 2012, and updated in December, 2012. Relevant reviews and meta-analyses of the role of sitagliptin, metformin and pioglitazone in β-cell function were examined for trials that might be included.

### Study selection

Clinical trials that investigated the role of glucose-lowering drugs (sitagliptin, metformin and pioglitazone) on β-cell function were eligible for inclusion. To be included in this meta-analysis, studies were required to: (1) be trials using sitagliptin, metformin or pioglitazone alone; (2) be trials initially using combined sitagliptin and metformin, metformin and pioglitazone, or sitagliptin and pioglitazone; (3) be placebo or active controlled; and (4) report HOMA-β and/or PI/IR data for patients with type 2 diabetes. Trials of combination therapy using one drug added to ongoing therapy were excluded.

### Data extraction and quality assessment

The quality of trials was assessed using some of the parameters proposed by Jadad et al. [10], which are used only for descriptive purposes. All studies were evaluated by three independent reviewers (Jin Lu, Jiajie Zang and Huihua Li) and disagreements were resolved by consensus.

Data extraction was conducted independently by two reviewers (Jin Lu and Jiajie Zang) using a standardized approach. Disagreements were adjudicated by a third reviewer (Huihua Li) after referring back to the original articles. We used a standardized data collection form to extract the following information: publication information (title, first author's name and year of publication), study characteristics (regimen details, duration of treatment, duration of diabetes and doses), characteristics of the study population (sample size and distribution of age and sex) and clinical outcomes (HOMA-β, PI/IR and HbA1c).

### Data synthesis and analysis

We used version 2 of the Comprehensive Meta Analysis software for all statistical analyses. For each study, we determined the change in β-cell function in patients with diabetes, with 95% confidence intervals (CIs). Data on the change from baseline of β-cell function with sitagliptin, metformin or pioglitazone alone and combined sitagliptin and metformin, metformin and pioglitazone or sitagliptin and pioglitazone were analyzed.

In each meta-analysis, *χ* ^2^ and *I* ^2^ values were first calculated to assess the heterogeneity of the included trials [11]; *P*<0.10 for the chi-square test and *I* ^2^<25% were interpreted as signifying low-level heterogeneity. Regardless of whether there is statistically significant heterogeneity, a pooled effect should be calculated using a random-effects model. To be more conservative, random-effects models were employed for all analyses. To calculate pooled changes of β-cell function, an inverse variance statistical method was used. We also assessed the probability of publication bias using funnel plots and Egger's test . Statistical significance was defined as a two-tailed *P* value of 0·05. The interaction test [12] was used to compare the effects of different drugs. Two-tailed *P*<0.05 was deemed statistically significant.

## Results

### Flow of included studies

**Figure 1:**
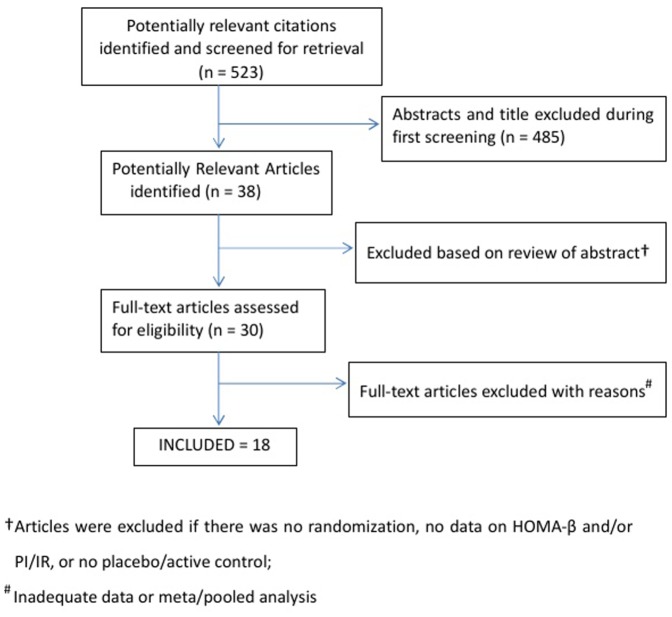
Results of the article search and outline of the process of searching for articles for this meta-analysis.

The study selection process is described in Figure 1. Of 523 screened articles and abstracts, 38 relevant articles and abstracts were identified, 30 were retrieved for detailed evaluation and 18 [13]–[30] met the inclusion criteria. Seventeen studies provided data adequate for meta-analysis of HOMA-β (*n* = 8901) and 13 for PI/IR (*n* = 7236). All included trials described the method of randomization and blinding.

### Study characteristics

Table 1 shows the characteristics of the individual trials. All included studies used metformin 1000 mg/d, sitagliptin 100 mg/d or pioglitazone 30–45 mg/d for monotherapy or combination therapy. All studies reported HOMA-β and/or PI/IR for 12–54 weeks. The mean age of the study participants did not vary greatly between the individual studies and averaged 56 years. Across all studies, there were slightly more men than women in a predominantly white patient population, though two studies were performed in Japanese patients. Average baseline HbA1c ranged from 7.5% (58 mmol/mol) to 9.0% (75 mmol/mol), but two studies reported patients with average baseline HbA1c ranging from 9.5% (80 mmol/mol) to 10.5% (91 mmol/mol).The average duration of known diabetes was 4.71 years.

**Table 1:** Characteristics of relevant primary studies.

Study	Design/Dose	Number	Study duration (wk)	Mean age (y)	Gender (M %)	Duration of known diabetes (y)	Baseline HbA1c (%)	Baseline HOMAβ (%)
004(13)	Metformin 1000 mg bid	137	54	54.2	45	4.1	8.5	44.3
	Sitagliptin 100 mg qd	106	54	53.5	52	3.9	8.7	40.8
	Sitagliptin+metformin	157	54	53.6	41	4.6	8.7	44.0
011(14)	Metformin 1000 mg bid	177	24	NR	NR	4.5	8.68	44.8
	Sitagliptin 100 mg qd	175	24	NR	NR	4.5	8.87	37.9
	Sitagliptin+metformin	178	24	NR	NR	4.5	8.76	41.4
016(15)	Metformin 1000 mg bid	522	24	55.7	44	2.1	7.2	90.5
	Sitagliptin 100 mg qd	528	24	56.3	48	2.6	7.2	80.2
017(16)	Metformin 1000 mg bid	621	18	50	57	3.2	9.8	62.4
	Sitagliptin+metformin	625	18	49.4	56	3.5	9.9	50.5
005(17)	Sitagliptin 100 mg qd	124	12	55.1	52.4	4.2	7.83	49.8
007(18)	Sitagliptin 100 mg qd	234	24	NR	NR	4.4	8.01	57.6
008(19)	Sitagliptin 100 mg qd	201	18	NR	NR	4.5	8.04	53.3
009(20)	Sitagliptin 100 mg qd	110	12	56	55.5	3.6	7.78	63.2
014(21)	Sitagliptin 100 mg qd	75	12	55.6	60	4	7.54	31.0
002(22)	Pioglitazone 30–45 mg qd	256	28	52.2	52.3	3.3	8.9	52.1
	Sitagliptin+metformin	261	28	52.4	54.8	3.2	8.9	56.1
020(23)	Pioglitazone 30–45 mg qd	19	12	61.4	73.7	2.6	6.7	71.6
022(24)	Pioglitazone 30–45 mg qd	54	26	60.6	57.4	11.3	7.8	NR
024(25)	Pioglitazone 30–45 mg qd	120	12	52.6	70.8	0*	10.1	NR
019(26)	Sitagliptin+metformin	150	24	54.9	59.3	6.1	8.7	50.3
003(27)	Metformin+pioglitazone	76	48	58	51.3	6	8.4	52.1
003(27)	Sitagliptin+pioglitazone	75	48	57	49.3	5	8.5	54.6
013(28)	Metformin+pioglitazone	87	18w	54.8	63	4.6	7.73	62.6
023(29)	Metformin+pioglitazone	142	28	59.2	66.2	12.1	8.2	163.7
001(30)	Sitagliptin+pioglitazone	175	24w	55.6	53.1	6.1	8.05	36.2

NR: not reported.

### Effect of monotherapies on HOMA-β

**Figure 2:**
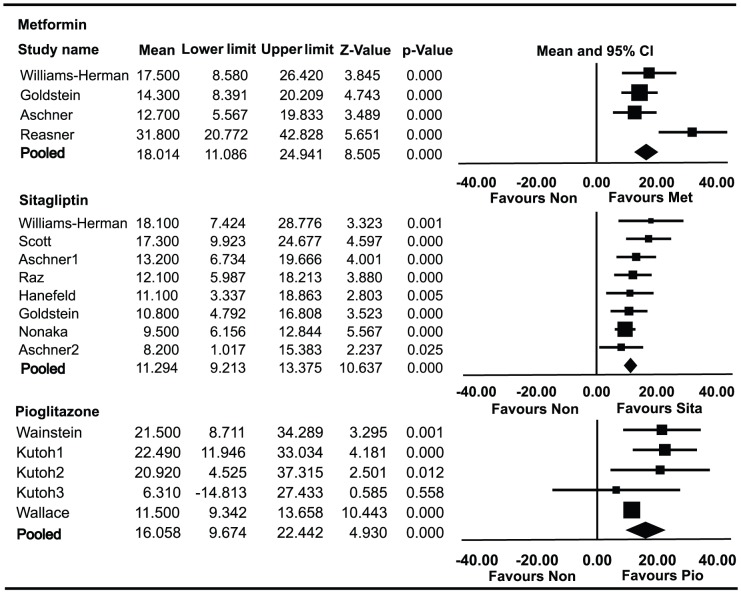
Meta-analyses for changes in the homeostasis model assessment of β-cell (HOMA-β) for different monotherapies. Favours Non : favours no medication.

Data on HOMA-β in patients using metformin alone were available for 1,457 patients enrolled in four trials (Figure 2). The absolute value of HOMA-β was elevated by 18.01% (95% CI 11.09% to 24.94%) from baseline. HbA1c decreased by 1.30% (95% CI −1.15% to −1.45%) from baseline. Data on HOMA-β in patients using sitagliptin alone were available for 1,553 patients enrolled in eight trials. The absolute value of HOMA-β was elevated by 11.29% (95% CI 9.21% to 13.37%) from baseline. HbA1c decreased by 0.8% (95% CI −0.60% to −1.00%) from baseline. Data on HOMA-β in patients using pioglitazone alone were available for 395 patients enrolled in three trials. The absolute value of HOMA-β was elevated by 16.06% (95% CI 9.67% to 22.44%) from baseline. HbA1c decreased by 1.29% (95% CI −0.54% to −2.03%) from baseline.

HOMA-β and HbA1c were significantly improved in patients who received metformin monotherapy compared with sitagliptin (*P* = 0.040, *P* = 0.0006). However, the changes of HOMA-β and HbA1c with metformin monotherapy were not statistically significantly different from those with pioglitazone (*P* = 0.699, *P* = 0.973).

### Effect of combined therapies on HOMA-β

**Figure 3:**
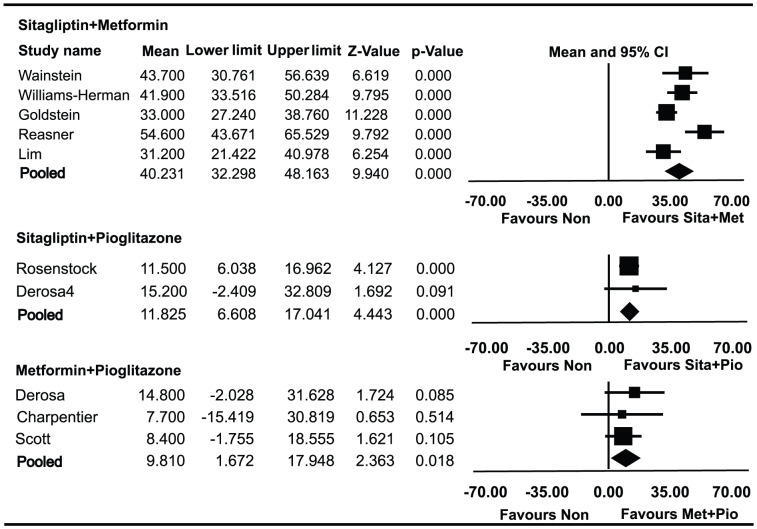
Meta-analyses for changes in the homeostasis model assessment of β-cell (HOMA-β) for different combined therapies. Favours Non : favours no medication.

Data on HOMA-β in patients using metformin and sitagliptin combined therapy were available for 1371 patients enrolled in five trials (Figure 3). The absolute value of HOMA-β was elevated by 40.23% (95% CI 32.30% to 48.16%) from baseline. HbA1c decreased by 1.78% (95% CI −1.49% to −2.07%) from baseline. Data on HOMA-β in patients using sitagliptin and pioglitazone combined therapy were available for 250 patients enrolled in two trials. The absolute value of HOMA-β was elevated by 11.82% (95%CI, 6.61% to 17.04%) from baseline. HbA1c decreased by 1.05% (95% CI, −0.36% to −1.73%) from baseline. Data on HOMA-β in patients using metformin and pioglitazone combined therapy were available for 305 patients enrolled in three trials. The absolute value of HOMA-β was elevated by 9.81% (95% CI 1.67% to 17.95%) from baseline. HbA1c decreased by 1.40% (95% CI −1.22% to −1.59%) from baseline.

HOMA-β was significantly improved in patients who received metformin and sitagliptin combined therapy compared with both sitagliptin and pioglitazone (*P* = 0.000) and metformin and pioglitazone (*P* = 0.022). HbA1c was significantly decreased in patients who received metformin and sitagliptin compared with metformin and pioglitazone (*P* = 0.026), but there was no statistically significant difference in the decrease of HbA1c between sitagliptin and metformin combined therapy and sitagliptin and pioglitazone (*P* = 0.195). There was no significant difference in the change of HOMA-β or HbA1c between sitagliptin and pioglitazone combined therapy and metformin and pioglitazone (*P* = 0.774, *P* = 0.473).

### Effect of monotherapies on PI/IR

**Figure 4:**
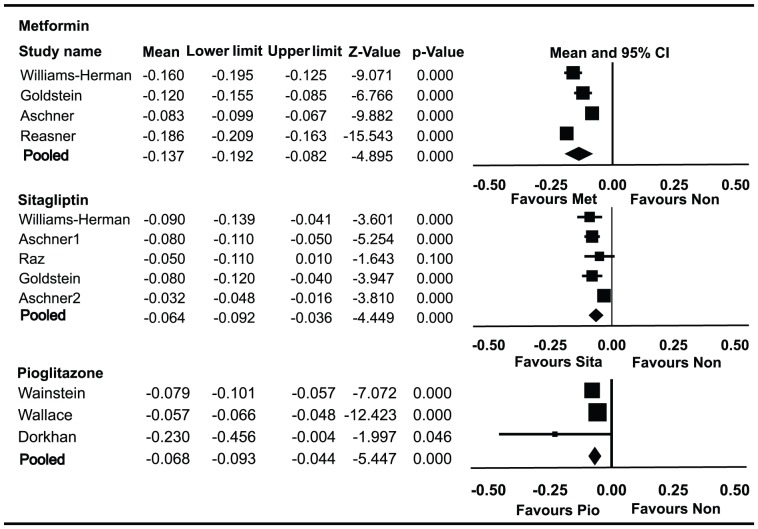
Meta-analyses for changes in the proinsulin/insulin ratio (PI/IR) for different monotherapies. Favours Non : favours no medication.

Data on PI/IR in patients using metformin alone were available for 1420 patients enrolled in four trials (Figure 4). PI/IR decreased by 0.137 (95% CI, −0.082 to −0.192) from baseline. HbA1c decreased by 1.30% (95% CI −1.15% to −1.45%) from baseline. Data on PI/IR in patients using sitagliptin alone were available for 1,199 patients enrolled in five trials. PI/IR decreased by 0.064 (95% CI −0.036 to −0.092) from baseline. HbA1c decreased by 0.80% (95% CI −0.60% to −1.00%) from baseline. Data on PI/IR in patients using pioglitazone alone were available for 329 patients enrolled in three trials. PI/IR decreased by 0.068 (95% CI −0.044 to −0.093) from baseline. HbA1c decreased by 1.06% (95% CI −0.17% to −1.95%) from baseline.

PI/IR was significantly improved in patients who received metformin monotherapy compared with sitagliptin (*P* = 0.019) or pioglitazone (*P* = 0.015). HbA1c was significantly improved in patients who received metformin monotherapy compared with sitagliptin (*P* = 0.0005), but there was no statistically significant difference in the decrease of HbA1c between metformin and pioglitazone monotherapy (*P* = 0.740).There was no significant difference in the decrease of PI/IR or HbA1c between sitagliptin and pioglitazone monotherapy (*P* = 0.656).

### Effect of combined therapies on PI/IR

**Figure 5:**
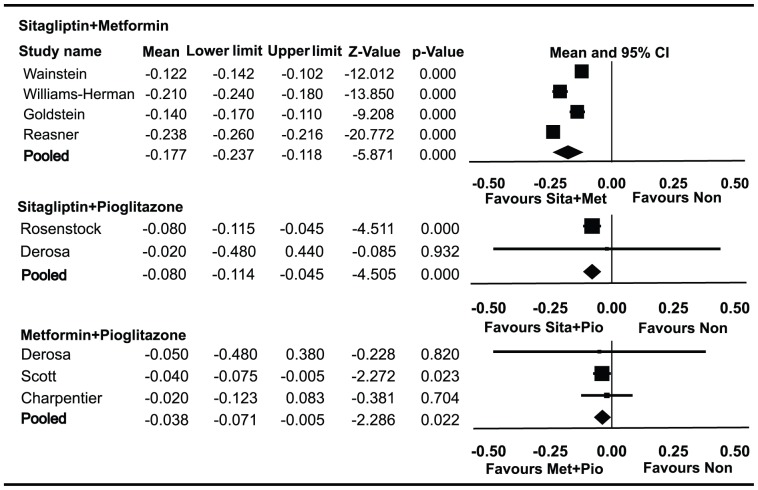
Meta-analyses for changes in the proinsulin/insulin ratio (PI/IR) for different combined therapies. Favours Non : favours no medication.

Data on PI/IR in patients using metformin and sitagliptin combined therapy were available for 1,199 patients enrolled in four trials (Figure 5). PI/IR decreased by 0.177 (95% CI −0.118 to −0.237) from baseline. HbA1c decreased by 1.90% (95% CI −1.75% to −2.05%) from baseline. Data on PI/IR in patients using sitagliptin and pioglitazone combined therapy were available for 250 patients enrolled in two trials. PI/IR decreased by 0.080 (95% CI −0.045 to −0.114) from baseline. HbA1c decreased by 1.12% (95% CI −0.58% to −1.66%) from baseline. Data on PI/IR in patients using metformin and pioglitazone combined therapy were available for 305 patients enrolled in three trials. PI/IR decreased by 0.038 (95% CI −0.005 to 0.071) from baseline. HbA1c decreased by 1.09% (95% CI −0.49% to −1.69) from baseline.

PI/IR was significantly improved in patients who received metformin and sitagliptin combined therapy compared with both sitagliptin and pioglitazone (*P* = 0.007) and metformin and pioglitazone (*P* = 0.023).There was no statistically significant difference in the decrease of PI/IR between sitagliptin and pioglitazone combined therapy and metformin and pioglitazone (*P* = 0.289). There were no significant differences in the decrease of HbA1c among the three combination therapies (all *P*>0.05).

All the subgroup analysis for β-cell function related to HOMA-β and PI/IR are listed in Table 2.

**Table 2:** Subgroup analysis for β-cell function related to HOMA-β and PI/IR.

Subgroup	No. studies	*N*	WMD	95%CI
HOMA-β (monotherapy)				
Metformin	4	1457	18.01%	(11.09%,24.94%)
Sitagliptin	8	1553	11.29%	(9.21%,13.37%)
Pioglitazone	3	395	16.06%	(9.67%,22.44%)
HOMA-β (combination therapy)				
Metformin+sitagliptin	5	1371	40.23%	(32.30%,48.16%)
Metformin+pioglitazone	3	305	9.81%	(1.67%,17.95%)
Sitagliptin+pioglitazone	2	250	11.82%	(6.61%,17.04%)
PI/IR (monotherapy)				
Metformin	4	1420	−0.137	(−0.082,−0.192)
Sitagliptin	5	1199	−0.064	(−0.036,−0.092)
Pioglitazone	3	329	−0.068	(−0.044,−0.093)
PI/IR (combination therapy)				
Metformin+sitagliptin	4	1199	−0.177	(−0.118,−0.237)
Metformin+pioglitazone	3	305	−0.038	(−0.005,−0.071)
Sitagliptin+pioglitazone	2	250	−0.080	(−0.045,−0.114)

WMD: weighted mean difference; CI: confidence interval.

## Discussion

Reductions in HOMA-β have historically been used a measurement of β-cell deterioration and increases in HOMA-β are considered beneficial [7]. One mechanism of β-cell deterioration is reduction of β-cell mass. On autopsy of human pancreatic tissue, a significant reduction in β-cell mass was observed in individuals who had impaired glucose tolerance (40%) or type 2 diabetes (60%) compared with controls without diabetes [31]. Logically, inhibition of increased apoptosis, in light of normal islet neogenesis, may lead to the restoration of β-cell mass and thereby improvement of β-cell function [7].

Preclinical studies have suggested that chronic administration of sitagliptin increases β-cell mass, improves the ratio of insulin positive cells to islet area and restores β-cell to α-cell ratio [32]. Thiazolidinediones are peroxisome proliferator-activated receptor gamma agonists that regulate the expression of genes modifying cellular differentiation and glucose/lipid metabolism [33]. They have also been shown to enhance β-cell function in both animals and humans with diabetes and to preserve β-cell mass via stimulation of neogenesis [33]. However, the monotherapy subgroups of this meta-analysis indicate that HOMA-β significantly favors metformin over sitagliptin (18.01% vs. 11.29%). The improvement in HOMA-β in the metformin monotherapy subgroup was also higher than that in the pioglitazone subgroup (18.01% vs. 16.06%), but not significantly. There have been few studies of the effect of metformin on β-cell function, though some in vitro evidence implies that metformin may improve β-cell function and prevent β-cell apoptosis [34], [35]. The effect of metformin on β-cell function in vivo should to be investigated in future studies.

Reduction in β-cell mass may also occur because of β-cell injury accelerated by prolonged exposure to glucotoxicity [36]. In this meta-analysis, a significant improvement in HbA1c was observed in the metformin monotherapy subgroup compared with the sitagliptin subgroup (1.30% vs. 0.8%). It is plausible that the improved HOMA-β achieved by metformin may, in part, be associated with elevation of HbA1c. Obviously, the mechanisms of glycemic durability signify the inadequacy of HOMA-β as an independent measure of β-cell function.

HOMA-β cannot be used accurately when the β-cells are stressed to such an extent that intact proinsulin is secreted together with or instead of insulin in the fasting state (stage III of β-cell dysfunction) [37]. Proinsulin is also able to lower glucose levels, but is not considered in the HOMA equations. Measurement of fasting intact proinsulin has been shown to be a very specific indicator of late stage β-cell dysfunction [37], so it is plausible that an improved PI/IR may, in part, be responsible for the difference in β-cell preservation and regeneration observed in preclinical studies.

In this meta-analysis, PI/IR decreased after monotherapy with metformin, sitagliptin and pioglitazone, indicating that all three drugs preserve β-cells. PI/IR significantly favored metformin over sitagliptin and pioglitazine, even in the presence of an equivalent reduction in HbA1c. Other mechanisms independent of glucotoxicity may play a role in the improvement of PI/IR with metformin.

Decreases in PI/IR may possibly be related to the resistance profile of the β-cells [3]–[5]. Recently, it has been suggested that elevated proinsulin levels are independently associated with insulin resistance, possibly as a result of insulin resistance at the islet cell level [5]. This may be the reason why PI/IR increases with the use of sulfonylureas but decreases with metformin, sitagliptin or pioglitazone. Whether metformin improves β-cell insulin resistance better than sitagliptin or pioglitazone should be interpreted with caution. Decreases in PI/IR indicate an improvement in the secretory ability of β-cells [3]–[5]. PI/IR is negatively correlated with insulin secretion in both healthy subjects and those with diabetes, implying that PI/IR may be a strong predictor of β-cell dysfunction [7].

A significant amount of pharmacological data generated using metformin has provided insights into the effects of acute activation of the AMPK pathway on insulin secretion. Early work on this pathway in β-cells aimed to evaluate the impact of maintaining its activity with acute AICAR treatment (i.e. throughout the duration of the insulin secretion assay). Perfusion of the pancreas with an intermediate concentration of glucose (6 mM) together with increasing doses of AICAR enhanced insulin secretion, suggesting that AMPK activation promotes insulin secretion [38]. Follow-up studies reported that AICAR treatment increased insulin secretion in cell lines and rat islets across a wide range of glucose concentrations [39].

Metformin combined with sitagliptin achieved more pronounced improvements in HOMA-β and PI/IR than any other combination therapy. Another possible mechanism of β-cell preservation is a decrease in apoptosis via increased glucagon-like peptide-1 (GLP-1) and gastric inhibitory polypeptide (GIP) signaling [40], [41]. Incidentally, when compared in a crossover study of healthy subjects, metformin increased active GLP-1 less than did sitagliptin, but the two drugs acted synergistically in combination [42]. Both HOMA-β and PI/IR were improved with metformin and synergistically with metformin and sitagliptin in our study, indicating complementary effects possibly because of increased GLP-1 activity. Studies have shown that GLP-1 treatment achieved dose-dependent prevention of apoptosis in a β-cell line in vitro [43]. GIP has also been reported to stimulate β-cell proliferation [43]. Further studies of cellular GLP-1 and GIP signaling are needed to elucidate this mechanism.

The limitations of this meta-analysis should be noted. Although metformin was shown to improve β-cell function more than both sitagliptin and pioglitazone, the study periods (ranging from 12 to 54 weeks) seem to be a little short and we were unable to determine the long term effect on β-cell survival. Longer, randomized trials evaluating changes of surrogate markers of β-cell function with metformin treatment compared with sitagliptin or pioglitazone are needed. Based on the above findings, longer, high quality, large sample size and multicenter RCTs will help to identify the most effective treatment option for patients with type 2 diabetes.

## Supporting Information

Checklist S1 PRISMA Checklist.
(DOC)
